# Validation of a SARS-CoV-2 Surrogate Neutralization Test Detecting Neutralizing Antibodies against the Major Variants of Concern

**DOI:** 10.3390/ijms241914965

**Published:** 2023-10-06

**Authors:** Eveline Santos da Silva, Jean-Yves Servais, Michel Kohnen, Vic Arendt, Therese Staub, the CON-VINCE Consortium, the CoVaLux Consortium, Rejko Krüger, Guy Fagherazzi, Paul Wilmes, Judith M. Hübschen, Markus Ollert, Danielle Perez-Bercoff, Carole Seguin-Devaux

**Affiliations:** 1 Department of Infection and Immunity, Luxembourg Institute of Health, 29 Rue Henri Koch, L-4354 Esch-sur-Alzette, Luxembourg; eveline.santosdasilva@lih.lu (E.S.d.S.); jean-yves.servais@lih.lu (J.-Y.S.); judith.huebschen@lih.lu (J.M.H.); markus.ollert@lih.lu (M.O.); danielle.perezbercoff@lih.lu (D.P.-B.); 2 National Service of Infectious Diseases, Centre Hospitalier de Luxembourg, 4 Rue Ernest Barblé, L-1210 Luxembourg, Luxembourg; kohnen.michel@chl.lu (M.K.); arendt.vic@chl.lu (V.A.); staub.therese@chl.lu (T.S.); 3 Transversal Translational Medicine, Luxembourg Institute of Health; Centre Hospitalier de Luxembourg, 4 rue Ernest Barblé, L-1210 Luxembourg, Luxembourg; rejko.krueger@lih.lu; 4 Translational Neuroscience, Luxembourg Centre for Systems Biomedicine, University of Luxembourg, 6 avenue du Swing, L-4367 Belvaux, Luxembourg; 5 Department of Precision Health, Luxembourg Institute of Health, 1AB Rue Thomas Edison, L-1445 Strassen, Luxembourg; guy.fagherazzi@lih.lu; 6 Systems Ecology Group, Luxembourg Centre for Systems Biomedicine, 7 Avenue des Hauts Fourneaux, L-4362 Esch-sur-Alzette, Luxembourg; paul.wilmes@uni.lu; 7 Department of Life Sciences and Medicine, Faculty of Science, Technology and Medicine, University of Luxembourg, 6, Avenue du Swing, L-4367 Belvaux, Luxembourg

**Keywords:** SARS-CoV-2, viral neutralization assay, immunity, neutralizing antibodies, vaccines

## Abstract

SARS-CoV-2 infection and/or vaccination elicit a broad range of neutralizing antibody responses against the different variants of concern (VOC). We established a new variant-adapted surrogate virus neutralization test (sVNT) and assessed the neutralization activity against the ancestral B.1 (WT) and VOC Delta, Omicron BA.1, BA.2, and BA.5. Analytical performances were compared against the respective VOC to the reference virus neutralization test (VNT) and two CE-IVD labeled kits using three different cohorts collected during the COVID-19 waves. Correlation analyses showed moderate to strong correlation for Omicron sub-variants (Spearman’s r = 0.7081 for BA.1, r = 0.7205 for BA.2, and r = 0.6042 for BA.5), and for WT (r = 0.8458) and Delta-sVNT (r = 0.8158), respectively. Comparison of the WT-sVNT performance with two CE-IVD kits, the “Icosagen SARS-CoV-2 Neutralizing Antibody ELISA kit” and the “Genscript cPass, kit” revealed an overall good correlation ranging from 0.8673 to −0.8773 and a midway profile between both commercial kits with 87.76% sensitivity and 90.48% clinical specificity. The BA.2-sVNT performance was similar to the BA.2 Genscript test. Finally, a correlation analysis revealed a strong association (r = 0.8583) between BA.5-sVNT and VNT sVNT using a double-vaccinated cohort (*n* = 100) and an Omicron-breakthrough infection cohort (*n* = 91). In conclusion, the sVNT allows for the efficient prediction of immune protection against the various VOCs.

## 1. Introduction

Multiple severe acute respiratory syndrome coronavirus 2 (SARS-CoV-2) variants have emerged since the beginning of the coronavirus disease 2019 (COVID-19) pandemic, resulting in multiple waves of COVID-19 infections [1,2]. The initial SARS-CoV-2 Wuhan strain evolved into new, emerging variants carrying different sets of mutations. Vaccines, infection, and breakthrough infection (BTI, infection after vaccination) elicit a panel of immune responses that provide protection from infection. Unfortunately, this protection decreases over time [3,4,5]. A study carried out during the Delta wave in Israel showed that in unvaccinated infected individuals, the adjusted rate of infection 4–6 months after infection was 10.5 per 100,000 person days at risk, and this rate increased by 3-fold over 12 months after infection. Among uninfected individuals who had received two doses of the mRNA vaccine, the rate was 21.1 within 2 months of the last dose, and it increased to 88.9 at 6 months [5]. Nevertheless, the Delta variant was quickly outcompeted by the highly mutated Omicron variants. The especially high number of mutations in the spike protein ensured viral evasion from pre-existing immunity acquired through infection or vaccination with earlier variants [3,6,7]. Interestingly, the protection against Omicron from two doses of vaccine fades by 4–6 months as well as after the booster (third dose), although less quickly [8]. Therefore, the waning of protective immunity and the emergence of new variants both result in an increase in infections and reinfections [5,9,10].

Humoral immunity plays a major role in the protection against SARS-CoV-2 infection; hence, antibody assessment is essential to guide vaccination strategies [11]. Although serological assays demonstrate high sensitivity and specificity in detecting IgG antibodies against SARS-CoV-2, the presence of IgG antibodies does not always correspond to neutralizing antibodies (NAb) [12,13]. In contrast, there is strong evidence that the presence of neutralizing antibodies is highly predictive of protective immunity [14,15,16]. Several studies demonstrated correlates of protection in vaccinated patients with spike antibody concentrations or neutralizing titers [17,18,19,20]. During the efficacy clinical trials of the Moderna mRNA vaccine and the Astra Zeneca ChadOx1 NCoV19 vaccine, concentrations and titers were inversely correlated with the risk of COVID-19 infection [21] or a reduced risk of symptomatic infection, with the neutralizing titer directly related to the vaccine efficacy [22,23].

The viral spike protein is the primary target of neutralizing antibodies, as it interacts with the angiotensin-converting enzyme 2 (ACE2) receptor on host cells, mediating viral entry into target cells [24,25]. The spike protein is a trimer composed of two subunits: the S1 subunit (comprising the N-terminal domain (NTD), the receptor-binding domain (RBD), and other subdomains), and the S2 subunit. The whole-virus neutralization test (VNT) is the golden standard for assessing neutralizing antibody titers in patient serum, reported as the reciprocal dilution of serum required to inhibit 50% of infection (NT50). The major drawback of this test is the requirement of a biosafety level 3 (BSL-3) facility and the fact that it is also labor-intensive and time-consuming. Several alternative methods have been developed since the beginning of the pandemic, such as the pseudotype-based VNT requiring pseudotyped whole virus and cells in a BSL-2 laboratory for viral stock preparation and titration with a delay for processing of 3–4 days, as the whole-virus-based assay, or surrogate tests in enzyme-linked immunosorbent assays (ELISA) format that can be performed in a BSL-1 laboratory and translated as an automated assay in a few hours. ELISA-based tests are virus- and cell-free and provide results in a few hours. Most commercially available surrogate neutralization tests detect antibodies that competitively hinder the interaction between purified recombinant ACE2 and the RBD, which has been primarily identified as the target of the majority of SARS-CoV-2 neutralizing antibodies [26]. Nonetheless, the S1 domain, the trimeric spike, and the S2 subunit can also be targeted by neutralizing antibodies [27]. Several studies have highlighted heterogeneous SARS-CoV-2 antibody responses upon infection or vaccination [28,29,30,31,32]. Taken together, these data highlight the need to detect a broad range of antibodies elicited by vaccination and infection [32].

Most surrogate tests detect antibodies targeting the RBD, and they were developed, evaluated, and validated against the Wuhan SARS-CoV-2 virus (B.1 strain) [33,34,35,36,37,38]. Little is known about the performance of SARS-CoV-2 surrogate test efficacy across a panel of VOCs. In light of the extensive accumulation of mutations in the RBD of Omicron variants [39], it is not surprising that antibodies targeting this region cannot be detected in surrogate tests based on ancestral spike proteins, as described in a report that revealed reduced sensitivity of commercial assays after primary infection with Omicron [40]. Many surrogate tests only target RBD-specific antibodies, leaving out NAb that bind other regions of the SARS-CoV-2 spike [34,35,41]. A recent study found a moderate correlation between surrogate and whole-virus test with BA.1 and BA.2 (Spearman’s r = 0.77 and 0.79 for BA.1 and BA.2, respectively) [42]. Few studies have investigated the accuracy of VOC-specific surrogates and correlated the results with VOC-specific neutralization titers quantified by reference whole-virus assay requiring a level 3 Biosafety Laboratory [40,42,43]. Consequently, we established a new surrogate test adapted to VOCs (B.1, Delta, Omicron BA.1, BA.2, and BA.5). This ELISA-based surrogate neutralization test (sVNT) is based on a trimeric spike for the detection of neutralizing antibodies that bind any domain of the SARS-CoV-2 spike and prevent the interaction with the ACE2 protein. The analytical performances of the assays were compared to the reference VNT using three different cohorts collected gradually during the emergence of the VOCs: an initial cohort, a previously published cohort [44], comprising sera from B.1-infected unvaccinated individuals (convalescent) collected in 2020 and sera from individuals who received a third dose of vaccine in 2021 (booster), then validated with a double-vaccinated cohort (samples from individuals vaccinated with two doses of vaccine in 2021), and a BTI cohort infected with Omicron variants in 2022. Our results clearly show that the sVNT nicely recapitulated the VNT based on whole virus for vaccinee samples, highlighting the usefulness of such easy-to-use tools to follow vaccine-induced humoral responses. In addition, the sVNT performed comparably to two CE-IVD-approved commercial kits, namely the Icosagen SARS-CoV-2 Neutralizing Antibody ELISA kit and the Genscript cPass kit, for the B.1 parental strain and Omicron BA.2.

## 2. Results

### 2.1. Establishment of Variant-Specific Surrogate Neutralization Tests (VOC-sVNTs)

**Figure 1:**
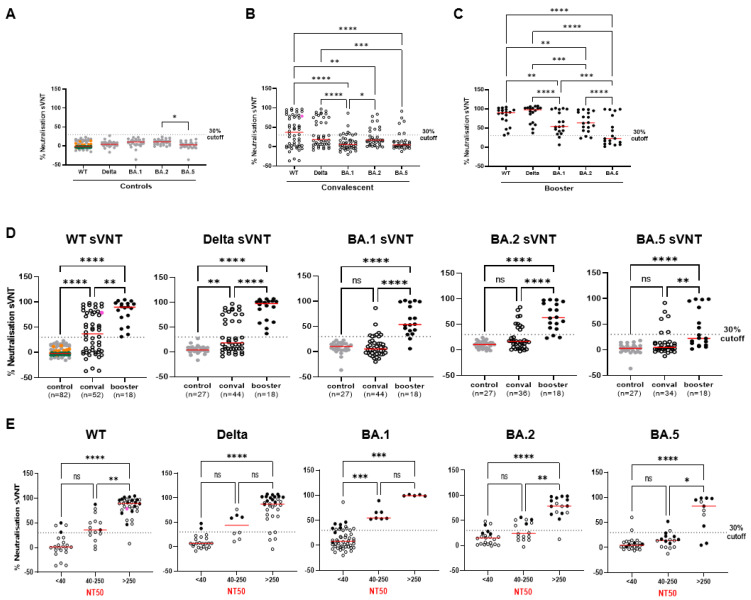
Dot plot of neutralization activity assessed by sVNT against WT SARS-CoV-2, Delta, and Omicron BA.1, BA.2, and BA.5 using control samples collected before 2019 (**A**), as well as samples from SARS-CoV-2-infected patients (**B**), and samples from individuals having received the booster (**C**). Comparison of sVNT results of control, convalescent (conval), and booster samples with each VOC as indicated above. The number (*n*) of tested samples is indicated below (**D**). Stratification of convalescent and booster sVNT results relative to NT50 titers assessed using the whole-virus assay (VNT). NT50 titers were grouped into low–no neutralization detected (samples with NT50 < 40), intermediate (samples with NT50 between 40 and 250), and highly neutralizing (samples with NT50 > 250) (**E**). Among the control samples, orange dots represent human Coronavirus (HCoV) sera panel (*n* = 20), while dark green (*n* = 35, HCV samples) and gray dots (*n* = 27, HIV-1 samples) indicate non-HCoV samples (**A**,**D**). Pink dot in WT-sVNT represents WHO International Standard (NIBSC code 20/136) (**B**,**D**–**E**). Open circles indicate convalescent samples, and closed circles indicate booster samples (**B**,**D**–**E**). The dotted lines indicate the 30% cut-off. Comparison analysis was performed using Kruskal–Wallis test with Dunn’s multiple comparisons test. *p*-values < 0.05 were considered significant; *, *p* < 0.05; **, *p* < 0.01; ***, *p* < 0.001; ****, *p* < 0.0001; ns, not significant.

We set-up an ELISA-based SARS-CoV-2 neutralization test where the ACE2 protein is coated to the ELISA plate, while clinical plasma samples are pre-incubated with a recombinant trimeric spike. Neutralizing antibodies will prevent the interaction of the trimeric spike with ACE2. We evaluated the performance of the sVNT in detecting neutralizing antibodies against the latest VOCs. As such, sVNT tests were carried out using either the SARS-CoV-2 B.1 spike containing the D614G mutation (termed WT), the Delta, Omicron BA.1, BA.2, or the BA.5 sub-variants’ trimeric spikes. Specificity of the WT-sVNT (i.e., sVNT performed using the WT trimeric spike) was firstly assessed using a human Coronavirus (HCoV) sera panel (*n* = 20) and non-HCoV samples (comprising 27 Human Immunodeficiency Virus (HIV) samples and 35 Hepatitis C Virus (HCV) samples) collected prior to 2019 (control samples, Table S1). All samples revealed no neutralization activity against the WT SARS-CoV-2 spike at the 1:10 dilution using a 30% cut-off (Figure 1A,D) previously used by other neutralization assays [36,45,46]. Additionally, specificity of Delta-, BA.1-, BA.2-, and BA.5-sVNT was assessed using non-HCoV samples (*n* = 27). All control samples were negative for each VOC sVNT under the 30% cut-off, indicating a specificity of 100% (Figure 1A).

To encompass a diverse range of immune profiles, we included clinical samples from unvaccinated patients infected by SARS-CoV-2 in 2020 (termed convalescent) (Figure 1B,D,E; see also Figure S1A–C and Table S1), as well as samples collected from individuals who received a third dose of the vaccine (termed booster) (Figure 1C–E; see also Figure S1A,B,D and Table S1). Clinical samples were tested using the WT spike, the Delta spike, and the Omicron spikes BA.1, BA.2, and BA.5, and the percent neutralization was calculated. As expected, the convalescent and booster samples achieved the highest neutralization activity against the WT and Delta spikes, while the neutralization activity against Omicron was significantly lower compared to WT or Delta (Figure 1B,C). Overall, the VOC-sVNT revealed that vaccine-induced antibodies in the booster samples harbored consistently significantly more neutralizing activity against each VOC spike compared to convalescent (Figure 1D).

We next compared the VOC-sVNT to the reference whole-virus neutralization test (VNT) using the WHO International Standard (NIBSC code 20/136), convalescent and booster samples against the whole-virus B.1 strain (WT), the Delta, and the Omicron sub-variants BA.1, BA.2, and BA.5 variants. VNT NT50 titers were determined and compared to the percentage of neutralization measured by sVNT. The results of the WHO standard were included in the convalescent group and showed a high neutralization activity in both tests: WT-sVNT had 78.7% neutralization activity, while the NT50 titers of the VNT reached 908 (pink dot in Figure 1B,D,E). Subsequently, the convalescent and booster samples were stratified based on NT50 titers measured using the VNT (NT50 ≤ 40, 40 < NT50 < 250, and NT50 ≥ 250). As shown in Figure 1E, the sVNT segregated low–no neutralization detected (NT50 < 40) and highly neutralizing sera (NT50 > 250) for all variants (Figure 1E).

Correlation analysis between sVNT and VNT confirmed low-to-good correlations for all VOCs sVNT (correlations ranging between 0.6042 and 0.8458) (Table 1 and Figure S1A). The highest correlation between sVNT and VNT was recorded for the WT (r = 0.8458) and Delta (r = 0.8158). Omicron sub-variants had lower correlation coefficients (r = 0.7081 for BA.1, r = 0.7205 for BA.2, and r = 0.6042 for BA.5) (Table 1 and Figure S1A). WT-sVNT, Delta-sVNT, and BA.1-sVNT provided an excellent performance in detecting neutralizing antibodies (AUC ranging from 0.9375 to 0.9900). In contrast, the AUC values for BA.2 and BA.5 were 0.8543 and 0.7746, respectively. Accordingly, using the 30% cut-off, sensitivities were 69.7 for BA.2 and 42.9% for BA.5. The poor sensitivity of the sVNT for the BA.2 and BA.5 sub-variants was mainly due to intermediate samples (Table 1 and Figure 1E). Evaluation of convalescent and booster samples separately against the different VOCs revealed that poor prediction capacities of the BA.2-sVNT and BA.5-sVNT concerned convalescent sera with an AUC of 0.8096 and 0.6321, respectively (Table 1 and Figure S1C). However, the neutralization activity of booster samples was readily detected and correctly identified in each VOC-sVNT. The AUC for booster samples evaluated against each VOC-sVNT resulted in an excellent performance (0.9375 for WT-sVNT, 1.000 for Delta-sVNT, 1.000 for BA.1, 1.000 for BA.2-sVNT, and 0.9821 for BA.5-sVNT) and at the 30% cut-off, we found 100% sensitivity for each VOC, except for BA.5 (Table 1 and Figure S1D). In this case, a cut-off adjustment to 20% improved the sensitivity of the BA.5-sVNT with booster samples (71% sensitivity). In summary, all the surrogate tests were able to accurately detect neutralizing antibodies in boosted individuals. The poor sensitivity of the BA.2-sVNT and BA.5-sVNT for first-wave convalescent sera may reflect the lesser neutralizing ability of infection-elicited antibodies against these highly evolved VOCs, as previously reported by us [47] and others [48,49].

### 2.2. Comparison of the WT-sVNT to Commercially Available Surrogate Tests

**Figure 2:**
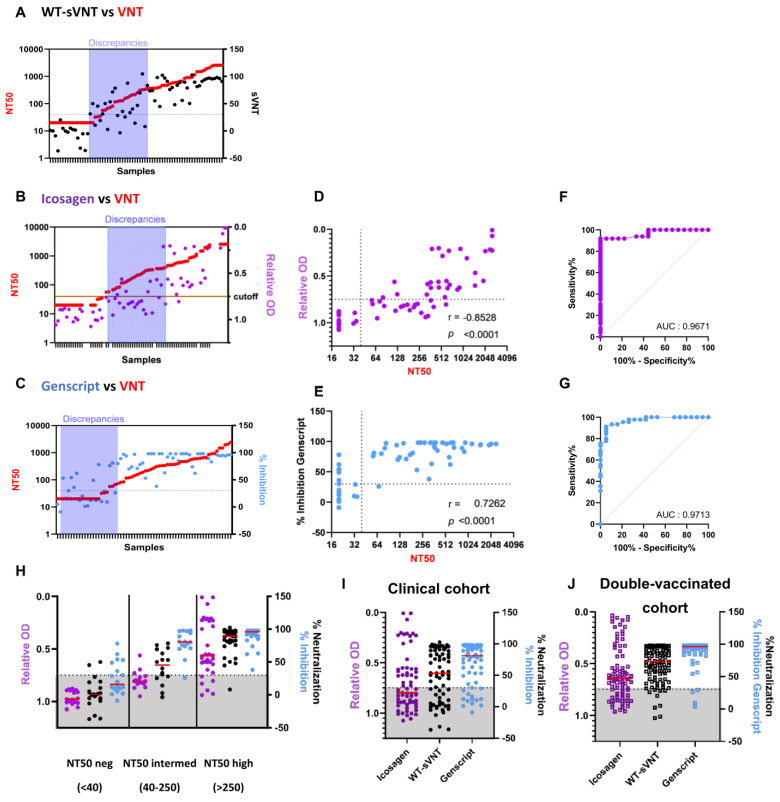
Comparison of WT-sVNT, Icosagen, and Genscript to whole-virus neutralization test with the WT strain. Sorting of clinical samples in regard to their NT50 results (in red) and their neutralizing activity assessed by WT-sVNT (in black, (**A**)), Icosagen (in purple, (**B**)), and Genscript cPass (in blue, (**C**)). Correlation analysis between Icosagen Relative OD and NT50 (**D**) and Genscript %Inhibition and NT50 (**E**). Spearman correlation coefficient (r) and *p*-value are indicated (**D**,**E**). ROC curve analysis of Icosagen (**F**) and Genscript cPass (**G**). AUC for each curve is indicated (**F**,**G**). Stratification of Icosagen results (relative OD, left Y-axis), Genscript and WT-sVNT assessments (%Inhibition and %Neutralization, respectively) on the right axis in regard to NT50 titers grouped into NT50neg (samples with NT50 below 40), NT50intermed (samples with NT50 between 40 and 250), and NT50high (samples with NT50 above 250) (**H**). Comparison of Icosagen, WT-sVNT, and Genscript assessments in clinical cohort (**I**) and in double-vaccinated cohort (**J**). The color code (sVNT = black; Icosagen = purple; Genscript = blue) is maintained throughout the text. Open squares with the corresponding color code indicate data collected from double-vaccinated cohort (**J**). The cut-offs indicated by a dotted line were set at 30% for Genscript and sVNT and 0.75 relative OD for Icosagen (**A**–**E**,**H**–**J**). Gray area indicates low–no neutralization detected samples below the cut-offs for all surrogate tests (**H**–**J**).

We next compared the sVNT to commercially available ELISA-based surrogate neutralization kits. Since these commercial kits were validated using the WT strain, we compared the WT-sVNT to the Genscript cPass kit and the Icosagen SARS-CoV-2 Neutralizing Antibody ELISA kit using the same clinical samples (convalescent and booster) (Figure 1E). The results were compared to the WT whole-virus neutralization test (VNT) to investigate potential discrepancies. We found that all surrogate tests had similar trends compared to the VNT (Figure 2A–C). Discrepancies between WT-sVNT and the whole-virus test were observed in samples showing low-to-intermediate neutralization (NT50) titers (Figure 1E and Figure 2A,H, and see Figure S1A). The Icosagen assay showed similar discrepancies for sera with low NT50 titers (Figure 2B,D,H). In contrast, the Genscript cPass test presented discrepancies for samples with low–no neutralization activity (NT50 < 40), thus resulting in considerably more false positive results (Figure 2C,E,H). Nonetheless, correlations between the neutralization activity assessed by surrogate tests and the whole-virus NT50 were good, ranging from 0.7262 for the Genscript cPass to −0.8528 for the Icosagen kit, while the WT-sVNT harbored an interesting midway profile (r = 0.8458, *p* < 0.0001) (Table 1 and Table 2 and Figure 2D–E, and see Figure S1A). Consequently, the ROC curve analysis reported that all three surrogate tests achieved an excellent performance in detecting neutralizing antibodies against the WT strain. The area under the curve (AUC) was almost identical for all three tests (WT-sVNT AUC reached 0.9495, Icosagen AUC reached 0.9671, and Genscript AUC reached 0.9713), indicating a high performance of our sVNT (Table 1 and Table 2 and Figure 2F,G, and see Figure S1B).

We next stratified the surrogate results by the VNT results (expressed in NT50). All low–no neutralization detected samples (NT50 < 40 according to the whole-virus-based assay) were qualified as low–no neutralization detected by Icosagen. Of these negative samples, two were identified as neutralizing with the WT-sVNT and seven with the Genscript cPass (Figure 2H). Conversely, analysis of whole-virus-confirmed highly neutralizing samples (NT50high) revealed no major difference between Genscript cPass and the WT-sVNT; Genscript cPass correctly assigned all NT50 high samples, while the WT-sVNT missed one sample and the Icosagen kit wrongly assigned six samples (Figure 2H). In line with the observations described above, most disparate assessments were recorded for samples with intermediate NT50 (NT50intermed); most samples were assessed as low–no neutralization detected by the Icosagen test, while most were considered neutralizing in the Genscript test. The WT-sVNT reported a midway profile with some neutralizing and some low–no neutralization detected results. Six out of those eight discordant samples in the WT-sVNT were similarly classified by the Icosagen or Genscript cPass tests, suggesting that neutralization assays based on whole-virus and surrogate tests detect distinct paratopes/antibodies (Figure S2A). Samples with intermediate NT50 resulted in less reliable assessments by all three surrogate tests.

We noticed a good correlation between the three ELISA-based tests (WT-sVNT and Genscript cPass revealed a Spearman’s correlation r of 0.8673 and WT-sVNT and Icosagen revealed a Spearman’s coefficient r of −0.8773), while the correlation between both commercial kits was lower (r = −0.7877), confirming the midway profile of the WT-sVNT (Table 2 and Figure S2B). We also observed major differences between the results, as hinted at in Figure 2H. Indeed, 37 out of 67 clinical samples (55.2%) assessed using the Icosagen kit were classified as low–no neutralization detected using the manufacturer’s 0.75 relative OD as the cut-off (Figure 2B,I and Table 2). Overall, at the 30% cut-off, WT-sVNT demonstrated acceptable specificity (close to Icosagen) and sensitivity (close to Genscript cPass) (Table 2).

To validate our findings, we tested an additional cohort composed of sera from individuals vaccinated with two doses of mRNA vaccine between 2020 and 2021 (referred as the double-vaccinated cohort, Table S1). Similarly to the results obtained with the initial clinical cohort (Figure 2I), we found major differences between all three surrogate tests: 27 samples out of 87 samples assessed by the Icosagen kit were classified as non-neutralizing samples (31%). The Genscript cPass test only reported 4 samples below the 30% cut-off out of 92 (4.3%), while the WT-sVNT reported 9 samples below the 30% cut-off out of 100 samples tested (9%) (Figure 2J). These data confirm the valuable midway profile of the WT-sVNT witnessed in the previous analysis (Figure 2H,I). Furthermore, the correlations between the tests were almost identical in the clinical cohort and in the vaccinated cohort, confirming the robustness of the WT-sVNT (Figure S2B,C).

### 2.3. The Ability of VOC-sVNT to Assess Neutralizing Antibodies against Omicron

**Figure 3:**
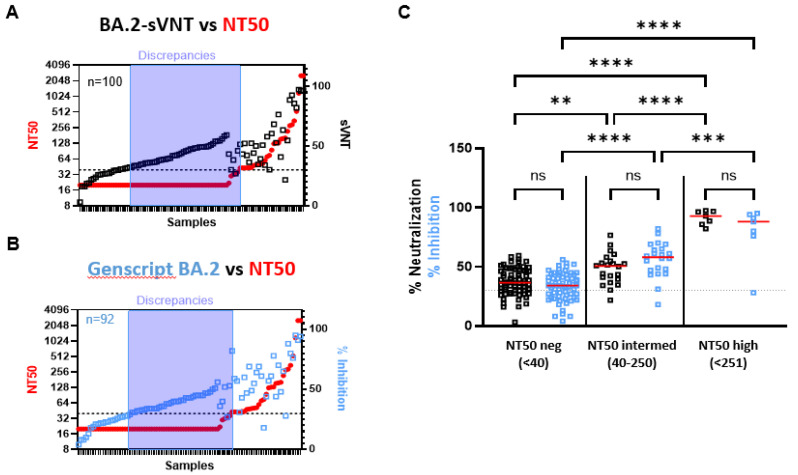
Evaluation of Omicron BA.2-specific surrogate tests. Performance of BA.2-sVNT (**A**) and Genscript cPass supplemented with BA.2 RBD (**B**) in double-vaccinated individuals (vaccinated cohort) in comparison to VNT reported in NT50. Sorting of vaccinated samples in regard to their NT50 results (in red on the left Y-axis) and their neutralizing activity assessed by BA.2-sVNT (in black, (**A**)) and Genscript-BA.2 (in blue, (**B**)) on the right Y-axis. Stratification of BA.2-sVNT and Genscript BA.2 results (%Neutralization and %Inhibition, respectively) in regard to NT50 titers grouped into NT50neg (samples with NT50 below 40), NT50intermed (samples with NT50 between 40 and 250), and NT50high (samples with NT50 above 250). A Kruskal–Wallis test followed by a Dunn’s multiple comparison post hoc test was used for comparisons between three groups (**C**). The BA.2-sVNT results are in black, and the Genscript-BA.2 results are in blue *p*-values < 0.05 were considered significant. **: *p* < 0.01; ***: *p* < 0.001. ****: *p* < 0.0001.

Waning immune responses and Omicron’s potential to escape vaccine-induced humoral immunity has been extensively described [50,51]. In this regard, it is of the upmost importance to accurately assess the presence of neutralizing antibodies against Omicron and its sub-variants. At the time of this work, there was no ready-to-use Omicron-specific surrogate test available from the Icosagen Company. Since most of the newly emerging variants are derived from BA.2 and BA.5, we set out to compare the BA.2-sVNT to the Genscript cPass kit based on the BA.2 RBD and compared surrogate assessments to the whole-virus results using a double-vaccinated cohort (*n* = 100) collected in 2021. We found that both surrogate tests gave very similar results (Figure 3A–C). Most discrepancies between the BA.2-sVNT and the whole-virus test correspond to NT50 negative samples that were assigned positive by the surrogate tests (Figure 3A and Figure S3A), but an identical profile was observed with the Genscript kit supplemented with the BA.2 RBD (Figure 3B and Figure S3A). Both surrogate tests revealed excellent sensitivity (BA.2-sVNT = 96.5%; Genscript-BA.2 = 93.1%) (Figure S3A) but poor correlation with the whole-virus results and with each other (r ranged from 0.4775 to 0.6517) (Figure S3B,C). Overall, stratification of the surrogate results by NT50 titers revealed no significant differences between the BA.2-sVNT and Genscript BA.2 tests (Figure 3C). Significant differences were observed between low–no neutralization detected samples (NT50neg) and samples with intermediate or high NT50 titers, highlighting the efficient discriminating capacity of both surrogate tests (Figure 3C).

**Figure 4:**
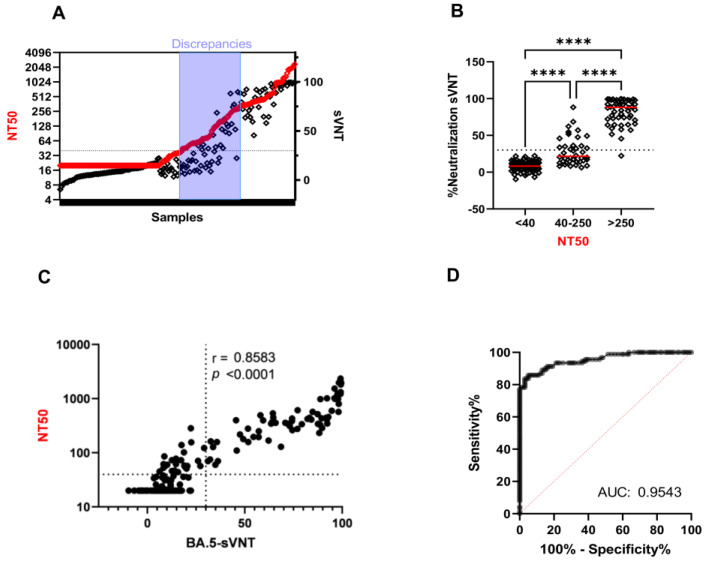
Performance of BA.5-sVNT in double-vaccinated and BTI samples (black diamond shapes) in comparison to BA.5 whole-virus neutralization test reported in NT50 (red diamond shapes) (**A**). Stratification of BA.5-sVNT results of vaccinated and BTI cohort in regard to NT50 titers grouped into NT50neg (samples with NT50 below 40), NT50intermed (samples with NT50 between 40 and 250), and NT50high (samples with NT50 above 250). A Kruskal–Wallis test followed by a Dunn’s multiple comparison post hoc test was used for comparisons between three groups (**B**). Correlation analysis between BA.5-sVNT and VNT. Spearman correlation coefficient (r) is indicated (**C**). ROC curve analysis of BA.5-sVNT with AUC (**D**). *p*-values < 0.05 were considered significant. ****: *p* < 0.0001.

Since many of the newly emerging variants are derived from the BA.5 sub-variant, we finally performed an in-depth investigation of the BA.5-sVNT with samples from double-vaccinated individuals (vaccinated cohort, *n* = 100) and individuals infected after vaccination (BTI cohort, *n* = 91) during BA.2 and BA.5 waves. No commercial BA.5 surrogate test was available, hence the BA.5-sVNT was only compared to the whole-virus test (Figure 4A). As previously observed (Figure 1E), discrepancies between BA.5-sVNT and the whole-virus test were observed in samples showing low-to-intermediate neutralization titers (NT50 40-250) (Figure 4B). Correlation analysis between BA.5-sVNT and VNT revealed a good performance with a correlation coefficient (r) of 0.8583 (Figure 4C). The ROC curve analysis of the BA.5-sVNT further highlighted the excellent performance of the test with an AUC of 0.9543 (Figure 4D). At the 30% cut-off, sensitivity reached 73.91%. Re-evaluation of the optimal cut-off for the BA.5-sVNT resulted in a specificity of 94.95% and a sensitivity of 85.87% at a cut-off of 17% instead of 30%. A closer look at each cohort revealed that the correlation coefficient in the vaccinated cohort was low (r = 0.4755) (Figure S4A), and the AUC was acceptable (AUC = 0.8553) (Figure S4B). Because the double-vaccinated cohort displayed low NT50s against the BA.2- and BA.5-sVNT, these findings again emphasize the poor performance of the VOC-sVNT for samples with low/intermediate neutralizing activity. Accordingly, the BA.5-sVNT showed excellent performance with the BTI cohort and displayed a correlation coefficient of 0.9453 (Figure S4D) and an AUC of 0.9792 (Figure S4E). At the 30% cut-off, the sensitivity was 86.11%. The Youden Index reached 86.1, supporting the reliability of the test for the BA.5 variant.

## 3. Discussion

The adequate and accurate detection of protective immune responses conferred by SARS-CoV-2 infection and/or vaccination is highly pertinent to anticipate individual protection over time and adopt personalized vaccination strategies. The aim of the current work was to develop and validate VOC-specific sVNTs, which has not been assessed in any other works, and to verify whether the use of a full spike would improve the performance of such sVNTs in terms of sensitivity, specificity, and reliability/concordance with the whole-virus-based standard assay. We assumed that the sVNT might be robust enough to discriminate neutralization activities between differently exposed individuals and at different time points after exposure following infection and/or vaccination.

The VOC-adapted sVNT described in this study showed 100% specificity and an overall good correlation with the whole-virus NT50 titers (r = 0.8458 between the WT-sVNT and the VNT) using a previously published cohort of convalescent and individuals having received a booster. Similar correlation strengths were found in a recent study comparing four SARS-CoV-2 surrogate virus neutralization assays using plasma samples from vaccinated and convalescent individuals, ranging from 0.7152 for the TECOmedical SARS-CoV-2-AK surrogate neutralization test to r = 0.8300 for the Genscript cPass test [52]. Using the sVNT, we found that the neutralizing capacity against VOCs enhanced after the booster vaccination, which is in line with numerous studies [53,54]. We did not compare the sensitivity and specificity between the spike and the RBD in our sVNT in the current work, although we observed some discordant sVNT results in low-to-intermediate NT50 samples, suggesting that the use of the full spike does not allow better discrimination than the RBD or that antibodies other than the anti-RBD can block binding of the spike to ACE2. However, correlations and AUC estimates were considerably higher in the booster samples compared to convalescent samples (Figure 1). The sVNT achieved good sensitivity for individuals vaccinated with three doses for WT (100%) and VOCs (57–100%). Sensitivity was lower for convalescent sera. This may reflect higher antibody titers in vaccinated individuals or qualitative differences between infection-elicited and vaccine-elicited antibodies, or a combination of both [44,47]. Similar findings were described in a recent study that highlighted variations between convalescent and vaccinated cohorts in SARS-CoV-2 surrogate virus neutralization tests; both surrogate tests (EuroImmun and Genscript cPass) reported discordant results compared to the VNT titers [55]. Another study also observed more indeterminate detections in convalescent than in vaccinee samples, confirming heterogenous neutralization profiles [30]. We might speculate that heterogeneous neutralization profiles are derived from different exposure frames and timings between vaccinees and convalescent, possibly resulting in different affinity maturation of B cell populations.

Differences in the sensitivities of commercial sVNTs to detect low levels of NAbs have been reported previously [52]. To compare the performance of the sVNT to other ELISA-based sVNTs, we selected the Genscript cPass SARS-CoV-2 Neutralization Antibody Detection kit since it was one of the first FDA and CE-IVD marked kits and has been extensively studied [35,42,43,52,55]. We also compared the sVNT to the Icosagen SARS-CoV-2 Neutralizing Antibody ELISA kit because it uses a trimeric spike rather than the RBD, albeit in a different test format. We first utilized the initial clinical cohort containing B.1-infected samples to evaluate the two commercial kits and compared them to our WT-sVNT. We found moderate correlation to the whole-virus results. The Icosagen test showed high specificity but it lacked sensitivity (61.2%), and it falsely classified almost 40% of samples with neutralizing activity (NT50 ≥ 40) as low–no neutralization detected. This is related to the 1/50 dilution used in this assay (and chosen by the manufacturer for CE IVD labeling) as compared to the 1/10 dilution of the Genscript assay or our sVNT. A similar trend was reported in a recent study that evaluated the inhibition of the ACE2–trimeric spike interaction by vaccine-induced antibodies [56]. Intriguingly, we and others observed the opposite trend for the Genscript cPass test, which showed impressive sensitivity (97.8%) at the 30% cut-off but poor specificity (63.2%) (Table 2) [55,57,58]. Although many studies reported high sensitivity and high specificity for the Genscript cPass test, a limited range of neutralization activity was assessed. Indeed, some studies used healthy donor samples as well as samples collected 2–3 weeks post-vaccination (thus at the peak of neutralization activity) and found a sensitivity of 71.1–100% and a specificity of 94.6–100% [59,60]. Our results are in agreement with the study of Adams O. and colleagues who reported that the Genscript cPass test harbored a low specificity with a specificity of 0.288 for convalescent samples and 0.500 for vaccinee samples [55]. Importantly, our study showed that the WT-sVNT had a midway profile between both commercial kits.

In light of the current Omicron-centered pandemic, detection of Omicron-specific neutralizing antibodies could greatly improve the assessment of individual protection. We found identical trends in BA.2-specific NAbs between sVNT and Genscript. Although the VNT revealed a major drop in neutralizing antibodies directed against BA.2, as expected [47], both tests overestimated BA.2-specific neutralization activity. Evaluation of the BA.5-sVNT revealed a good correlation with the VNT (r = 0.8583) for the double-vaccinated and the BTI cohorts and a lower sensitivity for booster and convalescent sera. It has been shown that BTI provide increased protection against variants, including Omicron [61]. Analysis of the BTI cohort using the BA.5-sVNT or the whole-virus test revealed a large range of neutralizing antibodies. Eventually, BA.5-sVNT performance compared to the whole-virus test was excellent, with a correlation of 0.9453 and an AUC of 0.9792. At the 30% cut-off, clinical specificity reached 100% and sensitivity reached 73.91% (Figure 4).

Taken together, our results showed that the VOC-sVNTs have good diagnostic performances in comparison to the golden standard. There are some limitations to the test, like the low sensitivity of the assay for low-to-intermediate NT50 samples. Since the exact level of neutralizing antibodies that are protective against the infection or against severe COVID-19 is yet unknown, it would be of great interest to further investigate low-to-intermediate NT50 samples and determine vaccination guidelines. Nonetheless, the detection of highly neutralizing NAb was very efficient, thus providing accurate advice for vaccination strategies upon antibody waning. Analysis of the different cohorts (convalescent, booster, double-vaccinated, and BTI) provided clinical relevance to the sVNT results, even in the Omicron context using booster and BTI samples. Due to the lack of a sufficient sample volume, some of the neutralization tests could not be performed for all the samples. This constraint precludes direct side-by-side comparisons of WT and VOC sVNTs. Another drawback from our study is the lack of highly neutralizing samples against Omicron. For instance, while the AUC and the sensitivity results for BA.1-sVNT are extremely high, this originates from the lack of neutralizing antibodies. There is indeed extensive literature about the BA.1 escape capacity from neutralizing antibodies. Most of our clinical samples (convalescent or triple-vaccinated “booster”) were collected before the Omicron wave and do not achieve any neutralizing activity against Omicron. Too few samples efficiently neutralize BA.1, BA.2, and BA.5 to have an accurate assessment of the Omicron-sVNT sensitivity. However, if omicron neutralization is not detected by the sVNT, there are likely to be low levels of neutralization, which may warrant a booster vaccination. Thus, our sVNT provides important insights into potential booster necessity. In addition, we found excellent and robust performances compared to other commercial surrogate tests for BA.5 with double-vaccinated and with BTI samples. Ultimately, longitudinal serological studies evaluating a threshold of neutralization titers that would trigger revaccination following waning of the vaccine-induced antibodies should emerge to provide such correlates of protection against emerging variants of concern [16] and support decision-makers on the administration of new vaccination strategies and on the optimal period between vaccine doses [23].

## 4. Material and Methods

### 4.1. Sample Collection

The specificity of the VOC sVNT was tested on plasma samples from HIV-1- (*n* = 27) and HCV-infected patients (*n* = 35) collected before 2019 at the Centre Hospitalier of Luxembourg (CHL, National Research Ethics Committee approvals n° 201105/07 and n° 201407/11), as well as a human Coronavirus (HCoV) sera panel purchased from In.vent Diagnostics GMBH (Hennigsdorf, Germany) (reference DSPA 4.1.9.16.1). The panel comprised the following pre-pandemic common corona sera samples: HCoV-HKU1 (*n* = 5), HCoV-OC43 (*n* = 5), HCoV-NL63 (*n* = 5), and HCoV-229E (*n* = 5) (Table S1). The international WHO anti-SARS-CoV-2 immunoglobulin standard (code 20/136) developed by the National Institute for Biological Standards and Controls (NIBSC) containing pooled plasma from 11 individuals recovered from SARS-CoV-2 infection in 2020 was used as a reference in the WT-sVNT.

The sensitivity was first assessed using sera from individuals infected with SARS-CoV-2 in Luxembourg. Anonymized residual serum samples were collected at CHL in 2020 and 2021 (LIH Institutional Review Board approval n° 14718697-NeutraCoV) as previously described [44]. Only the year of sampling and the time between sampling and infection, or the latest dose of vaccine, were available. The following sera of the clinical cohort were used: sera from 51 unvaccinated patients (convalescent sera) collected in 2020 early after acute infection, and sera from 18 individuals who had received 3 doses of vaccine (booster sera) collected in 2021. The time elapsed between the 3rd dose and sampling varied between 15 days and 10 months (median 5.3 months, interquartile range (IQR) 3.8–6.8).

The VOC-sVNT was further validated using samples from two COVID-19 cohorts from Luxembourg. The CON-VINCE study is a population-based cohort study that recruited a representative sample of the Luxembourg population (National Research Ethics Committee approval n° 202004/01). Samples were collected from individuals vaccinated with two doses of vaccine (*n* = 100) between April 2020 and June 2021. The time elapsed between the 2nd dose and sampling varied between 1 and 3 months (median 1.8 months, IQR 1–2.4). The Predi-COVID study is an ongoing hybrid cohort of people with confirmed SARS-CoV-2 infection. Samples used in this work were collected at the time of acute illness from laboratory-confirmed COVID-19 cases, either with or without symptoms, between March 2022 and August 2022, at the time of Omicron BA.1 transition to BA. 2 and emergence of BA.5 (National Research Ethics Committee approval n° 202003/07). The resulting cohort was named BTI cohort (*n* = 91). All participants signed an informed consent form, and samples were pseudo-anonymized. The current study represents a secondary use of the samples and data collected in the two cohorts, duly authorized by the National Research Ethics Committee (202209/06). Reduced sample volume availability did not allow us to perform all tests on all samples (Table S1).

### 4.2. Surrogate Virus Neutralization Test (sVNT) with VOCs

The sVNT is based on a two-step process. Firstly, MaxiSORP ELISA plates were pre-incubated with 100 ng per well of human ACE2 (hACE2-050P, eEnzyme, Gaithersburg, MD, USA, in phosphate-buffered saline (PBS) overnight at 4 °C. After coating, the ACE2-coated plate was washed 3 times with washing buffer (PBS-Tween 0.05%, Roti^®^ Fair PBST 7.4 purchased from Carl Roth, Karlsruhe, Germany) and incubated with blocking buffer for 1h at room temperature on an orbital shaker at 300 rpm. Secondly, samples diluted 1:10 in 50 µL blocking buffer (PBS with 0.05% Tween and 3% Bovine Serum Albumine (BSA, Sigma-Aldrich, Overijse, Belgium, catalog number A7030-100G) were pre-incubated with 50 µL of His-tagged trimeric SARS-CoV-2 spike (AcroBiosystems, Newark, NJ, USA) at a concentration of 1 ng/µL at 37 °C for 30 min to allow the binding of the trimeric spike to spike-specific antibodies. The serum–spike mixture was added to the ACE2-coated plate and incubated for 1 h at room temperature at 300 rpm. Trimeric spikes bound to spike-specific antibodies are unable to bind to ACE2 and are washed away (3 washes) with washing buffer. The remaining ACE2-bound trimeric spike was detected using horseradish peroxidase (HRP)-tagged anti-His antibodies (Sigma-Aldrich, #A7058) diluted 1:1000 in blocking buffer. After 1 h of incubation, 100 µL of 3,3′,5,5′-tetramethylbenzidine (ThermoFisher, Dilbeek, Belgium) was added to each well and incubated for 5 min in the dark. The reaction was stopped by addition of 100 µL of 2M sulfuric acid. Optical density (OD) was measured at 450 nm using a SpectraMax iD3 reader (Molecular Devices, Ismaning, Germany). Binding inhibition between ACE2 and His-tagged trimeric spike was determined using the following formula: Neutralization (%) = (1 − (sample OD_450_/non-serum control OD_450_)) × 100. A value below 0 indicates there is no binding of antibodies with the spike. All samples were tested in duplicate, and controls were tested in single well. An internal quality control was included to check the validity of the run. The following His-tagged SARS-CoV-2 trimeric spikes were used in the VOC sVNT: WT spike containing the D614G mutation (SPN-C52H3, AcroBiosystems), Delta spike (SPN-C52He, AcroBiosystems), Omicron BA.1 spike (SPN-C52Hz, AcroBiosystems), Omicron BA.2 spike (SPN-C5223, AcroBiosystems), and Omicron BA.5 spike (SPN-C522e, AcroBiosystems). The sequences of the spikes are based on the ectodomain of SARS-CoV-2 spike protein, which contains AA Val 16—Pro 1213 (Genbank accession number QHD43416.1). Sequence information is provided for each variant in the technical sheet of the manufacturer. To set up the assay, different sera dilutions were tested with high or intermediate neutralizing samples indicating that 1:20 or higher dilutions lacked reproducibility and sensitivity. The dilution of 1:10 was selected in accordance with the dilution of the first CE IVD label kit, the Genscript cPass^TM^ SARS-CoV-2 Neutralization Antibody Detection kit (Genscript Biotech, Ryswick, The Netherlands).

### 4.3. Commercial ELISA-Based Surrogate Neutralization Tests

Two commercially available ELISA-based SARS-CoV-2 surrogate neutralization tests were assessed and compared to our sVNT and to the whole-virus SARS-CoV-2 (NT50). The Genscript cPass^TM^ SARS-CoV-2 Neutralization Antibody Detection kit was performed according to the manufacturer’s instructions. Briefly, samples were diluted 1:10 and pre-incubated with HRP-conjugated-WT SARS-CoV-2 RBD protein contained in the kit. After a 30 min incubation at 37 °C, the mixture was transferred to ACE2-coated microtiter plate wells for another 15 min at 37 °C. After addition of the substrate solution followed by the stop solution, optical density (OD) was measured at 450 nm using a SpectraMax iD3 reader. Binding inhibition between ACE2 and HRP-RBD was determined using the following formula: Genscript Inhibition (%) = (1 − (Sample OD_450_/Negative Control OD_450_)) × 100. Control samples were tested in duplicate, while samples were tested in single wells. The 30% cut-off was used to determine neutralization capacity; hence, samples with percentage of inhibition ≥ 30% were considered as neutralizing [36,45,46], as recommended by the manufacturer. The Genscript cPass kit supplemented with the BA.2 SARS-CoV-2 RBD-HRP (Catalog Z03741-20, Genscript, Ryswick, The Netherlands) was evaluated with the vaccinated cohort.

The second commercially available ELISA-based SARS-CoV-2 surrogate neutralization test used was the SARS-CoV-2 Neutralizing Antibody ELISA kit (Icosagen, Ossu, Estonia). The test was carried out following the manufacturer’s instructions. Briefly, samples were diluted 1:50 and incubated in trimeric WT spike-coated microtiter plate wells for 20 min. Subsequently, HRP-conjugated-ACE2 was added for another 30 min incubation. After addition of the substrate solution followed by the stop solution, optical density (OD) was measured at 450 nm using a SpectraMax iD3 reader. Neutralization activity was expressed as relative OD by dividing the sample’s OD values by the mean value of the three repeated samples without serum. Regarding results interpretation, relative OD ≥ 0.75 is considered low–no neutralization detected, while relative OD < 0.75 indicates detectable neutralizing antibodies, as recommended by the manufacturer.

### 4.4. SARS-CoV-2 Virus Isolation and Whole-Virus Neutralization Test (VNT)

SARS-CoV-2 strains were cultured in a Biosafety level 3 laboratory. WT SARS-CoV-2 B.1 strain containing the D614G mutation as well as SARS-CoV-2 VOCs (Delta and Omicron BA.1, BA.2, and BA.5) were isolated in Luxembourg as previously described [47]. Briefly, residual SARS-CoV-2 swabs were incubated with Vero-E6 cells in Dulbecco’s modified Eagle medium (DMEM) containing antibiotics and 2% fetal bovine serum (FBS). Virus-induced cytopathic effect (CPE) was monitored daily, and viral supernatant was used as viral stock. The whole-virus neutralization assay has been described previously [44]. Briefly, serial dilutions of heat-inactivated (30 min at 56 °C) samples were incubated with virus on Vero-E6 cells. Virus-only and virus-free conditions (positive and negative controls, respectively) were included in each neutralization test. After 72 h of incubation at 37 °C, virus-induced CPE was measured using the tetrazolium salt WST-8. Percent of cell survival was calculated relative to uninfected cells (virus-free condition). The half-maximal inhibitory concentration for serum was determined by using the log(inhibitor) vs. dose–response and 4-parameter with variable slope with the GraphPad Prism 5 software. VNT results were log-transformed and expressed as 50% neutralizing titer (NT50). Sera with no neutralizing activity at the lowest dilution tested (1:40) were considered to have low–no neutralization detected. The threshold of 250 was set to define highly neutralizing antibody titers according to Feng S and colleagues [22].

### 4.5. Statistical Analysis

Statistical analyses of whole-virus tests were previously performed using GraphPad Prism 5 software as described [23]. Statistical analyses of the surrogate tests and comparisons were performed using GraphPad Prism 9.5 software. A Wilcoxon signed-ranked test was used for comparisons between two groups, and a Kruskal–Wallis signed-rank test was followed by a Dunn’s post hoc test for comparisons of three or more groups. Correlation coefficients (r) between sVNT, NT50, and commercial kits were determined using Spearman’s rank correlation. The Youden index was calculated by adding specificity (%) and sensitivity (%), then subtracting 100 from this value. *p*-values < 0.05 were considered significant.

## 5. Conclusions

Our study has shed new light on surrogate test efficiency in detecting neutralizing antibodies against the various newly emerging variants, especially against the Omicron sub-variants BA.1, BA.2, and BA.5, in a wide range of vaccination/infection profiles. We assessed for the first time whether VOC-adapted sVNT assays were necessary and validated the ability of our sVNT to detect neutralizing antibodies against emerging variants. Our results showed that variant-adapted assays are accurate and discriminate the neutralizing titers of differently exposed individuals at different time points after exposure. Our format offers an interesting intermediary profile between the two CE-IVD-marked sVNT tests. Our data emphasize the importance of continuous assay development due to the extensive mutations acquired by new VOCs and the accuracy of the sVNT to evaluate protection in vaccinated individuals.

## Figures and Tables

**Table 1:** Overview of the performance of each VOC sVNT compared to the whole-virus assay (VNT) of the corresponding strain. The Spearman’s correlation coefficients, the area under the curve (AUC), sensitivity (%), positive predictive value (PPV in %), negative predictive value (NPV in %), and number of clinical samples tested in Figure 1 and Supplementary Figure S1 are reported. Separate evaluation of convalescent and booster samples in each VOC-sVNT. nd, not determined.

Test	r (*p*-Value)	AUC	Sensitivity (%)	PPV (%)	NPV (%)	Sample (*n*)
**sVNT-WT**	**0.8458 (*p* < 0.0001)**	**0.9495**	**87.8**	**91.3**	**75.0**	**70**
Convalescent	0.8771 (*p* < 0.0001)	0.9506	81.8	92.9	75.0	52
Booster	0.6418 (*p* = 0.0041)	0.9375	100	88.9	nd	18
**sVNT-Delta**	**0.8158 (*p* < 0.0001)**	**0.9375**	**77.5**	**93.9**	**69.0**	**62**
Convalescent	0.7941 (*p* < 0.0001)	0.9229	62.5	100	69.0	44
Booster	0.6788 (*p* = 0.002)	1.00	100	88.9	nd	18
**sVNT-BA.1**	**0.7081 (*p* < 0.0001)**	**0.9900**	**100**	**48.0**	**100**	**62**
Convalescent	0.3606 (*p* = 0.0162)	0.9767	100	11.1	100	44
Booster	0.9366 (*p* < 0.0001)	1.00	100	68.8	100	18
**sVNT-BA.2**	**0.7205 (*p* < 0.0001)**	**0.8543**	**69.7**	**92.0**	**65.5**	**54**
Convalescent	0.5330 (*p* = 0.0008)	0.8096	47.4	90	61.5	36
Booster	0.8994 (*p* < 0.0001)	1.00	100	93.3	100	18
**sVNT-BA.5**	**0.6042 (*p* < 0.0001)**	**0.7746**	**42.9**	**84.6**	**56.7**	**52**
Convalescent	0.3172 (*p* = 0.0721)	0.6321	28.6	66.7	64.3	34
Booster	0.9415 (*p* < 0.0001)	0.9821	57.1	100	36.4	18

**Table 2:** Performance of surrogate neutralization tests and assessment of Spearman’s correlation coefficients in regard to the WT whole-virus test (VNT), the area under the curve (AUC), sensitivity (%), specificity (%), Youden index, overall percentage of low–no neutralization detected samples (%), and number of clinical samples tested in Figure 2A–I and Supplementary Figure S2A,B. Spearman’s correlation coefficients and *p*-values between the three surrogate tests are indicated as correlation (r).

Test	r (*p*-Value)	AUC	Sensitivity (%)	Specificity (%)	Youden Index	Below Cut-off (%)	Sample (*n*)	Correlation (r)	
								Genscript	Icosagen
sVNT	0.8458 (*p* < 0.0001)	0.9495	87.8	85.7	73.5	35.7	70	0.8673 (*p* < 0.0001)	−0.8773 (*p* < 0.0001)
Genscript	0.7262 (*p* < 0.0001)	0.9713	97.8	63.2	61	20.3	64	-	−0.7877 (*p* < 0.0001)
Icosagen	−0.8528 (*p* < 0.0001)	0.9671	61.2	100	61.2	55.2	67	−0.7877 (*p* < 0.0001)	-

## Data Availability

The data have been uploaded in the Zenodo repository. The following dataset was generated: Santos da Silva, Eveline, Servais, Jean-Yves, Kohnen, Michel, Arendt, Vic, Staub, Therese, Krüger, Rejko, Fagherazzi, Guy, Ollert, Markus, Perez-Bercoff, Danielle, and Seguin-Devaux, and Carole (2023). Validation of a SARS-CoV-2 surrogate neutralization test detecting neutralizing antibodies against the major variants of concern (dataset). Zenodo. https://doi.org/10.5281/zenodo.7973938 (accessed on 8 June 2023).

## Supplementary Materials

The following supporting information can be downloaded at https://www.mdpi.com/article/10.3390/ijms241914965/s1.

## Appendix A

**Convince consortium:** Tamir Abdelrahman, Geeta Acharya, Gloria Aguayo, Pinar Alper, Wim Ammerlaan, Ariane Assele-Kama, Christelle Bahlawane, Katy Beaumont, Nadia Beaupain, Lucrèce Beckers, Camille Bellora, Fay Betsou, Sandie Boly, Dirk Brenner, Henry-Michel Cauchie, Eleftheria Charalambous, Emilie Charpentier, Estelle Coibion, Delphine Collart, Manuel Counson, Brian De Witt, Olivia Domingues, Claire Dording, Bianca Dragomir, Tessy Fautsch, Jean-Yves Ferrand, Ana Festas Lopes, Guillaume Fournier, Joëlle Véronique Fritz, Manon Gantenbein, Piotr Gawron, Laura Georges, Soumyabrata Ghosh, Enrico Glaab, Clarissa Gomes, Borja Gomez Ramos, Vyron Gorgogietas, Jérôme Graas, Valentin Groues, Wei Gu, Gael Hamot, Anne-Marie Hanff, Maxime Hansen, Linda Hansen, Lisa Hefele, Laurent Heirendt, Ahmed Hemedan, Estelle Henry, Margaux Henry, Eve Herkenne, Sascha Herzinger, Christiane Hilger, Laetitia Huiart, Alexander Hundt, Judith Hübschen, Gilles Iserentant, Philipp Jägi, Anne Kaysen, Stéphanie Kler, Alexey Kolodkin, Rejko Krüger, Pauline Lambert, Jacek Jaroslaw Lebioda, Sabine Lehmann, Marie Leick, Anja Leist, Morgane Lemaire, Andrew Lumley, Annika Lutz, João Manuel Loureiro, Monica Marchese, Tainà Marques, François Massart, Patrick May, Maura Minelli, Alessandra Mousel, Maeva Munsch, Sophie Mériaux, Friedrich Mühlschlegel, Mareike Neumann, Trang Nguyen, Beatrice Nicolai, Marc Paul O’Sullivan, Leslie Ogorzaly, Jochen Ohnmacht, Christiane Olesky, Markus Ollert, Venkata P. Satagopam, Claire Pauly, Laure Pauly, Lukas Pavelka, Christian Penny, Magali Perquin, Achilleas Pexaras, Marie France Pirard, Jean-Marc Plesseria, Guilherme Ramos Meyers, Armin Rauschenberger, Lucie Remark, Basile Rommes, Kirsten Rump, Estelle Sandt, Bruno Santos, Aurélie Sausy, Margaux Schmitt, Reinhard Schneider, Valerie Schröder, Alexandra Schweicher, Sneeha Seal, Jean-Yves Servais, Florian Simon, Amna Skrozic, Chantal Snoeck, Kate Sokolowska, Lara Stute, Hermann Thien, Noua Toukourou, Christophe Trefois, Johanna Trouet, Nguyen Trung, Jonathan Turner, Michel Vaillant, Daniela Valoura Esteves, Carlos Vega Moreno, Charlène Verschueren, Maharshi Vyas, Claus Vögele, Cécile Walczak, Xinhui Wang, Femke Wauters, and Tania Zamboni.

**CoVaLux consortium:** Alexander Hundt, Alexander Skupin, Alexandre Baron, Alexey Kolodkin, Amy Parrish, Anja Leist, Antonio Cosma, Arnaud D’Agostini, Anke Wienecke-Baldacchino, Aurélie Fischer, Bernadette Leners, Charles Benoy, Christelle Bahlawane, Christelle Nicolas, Claus Vögele, Dimitros Kyriakis, Dmitry Bulaev, Enzo Bieber, Feng Q. Hefeng, Guy Fagherazzi, Henry-Michel Cauchie, Hugo Allemand, Irina-Afrodita Balaur, Isabelle Ernens, Ivana Paccoud, Jean-Yves Ferrand, Jérôme Graas, Jochen Klucken, Jochen Ohnmacht, Joëlle Fritz, Jorge Goncalves, Katy Beaumont, Kirstin Khonyongwa, Laetitia Schramm, Laura Georges, Léo Gerard, Lu Zhang, Luigi de Giovani, Manon Gantenbein, Marc Suhrcke, Mark Ritzen, Markus Ollert, Maurane Rollet, Maximilian Fünfgeld, Mélanie Vausort, Michaël Schnell, Michel Vaillant, Milena Despotovic, Myriam Alexandre, Nolwenn Badier, Olivia Domingues, Paul Palazzi, Paul Wilmes, Paula-Cristina Lupu, Philippe Kayser, Piyapong Khumrin, Priyanka Mendon, Rafaëla Schober, Rejko Krüger, Robert Sega, Sabrina Deroo, Sabrina Møller Gregorio, Sabrina Saracino, Sébastien Torres, Soumyabrata Ghosh, Sylvianne Poncelet, Thi Van Trang Nguyen, Valerie Voorsluijs, Venkata Pardhasaradhi Satagopam, Wim Ammerlaan, and Zoi Mavroeidi.
