# Supplementary material for: Validation of a SARS-CoV-2 Surrogate Neutralization Test Detecting Neutralizing Antibodies against the Major Variants of Concern
Source: Int J Mol Sci. 2023 Oct 6;24(19):14965. doi: 10.3390/ijms241914965 (PMC10573711; doi:10.3390/ijms241914965)
Supplement: Supplementary file 1 [file ijms-24-14965-s001.zip › ijms-2580910-supplementary.pdf]

Supplementary materials

		WT-sVNT	Delta-sVNT	BA.1-sVNT	BA.2-sVNT	BA.5-sVNT	Icosagen	Genscript WT	Genscript BA.2
Non-HCoV controls		62	27	27	27	27	0	0	0
HCoV panel		20	0	0	0	0	0	0	0
WHO International Standard		1	0	0	0	0	0	0	0
Clinical cohort	Convalescent	51	44	44	36	34	49	50	0
	Booster	18	18	18	18	18	18	14	0
Double-vaccinated cohort		100	0	0	100	100	87	92	92
BTI cohort		0	0	0	0	91	0	0	0
**TOTAL**		**252**	**89**	**89**	**181**	**270**	**154**	**156**	**92**

Supplement Table S1. Overview of the samples tested in each sVNT and commercial test.

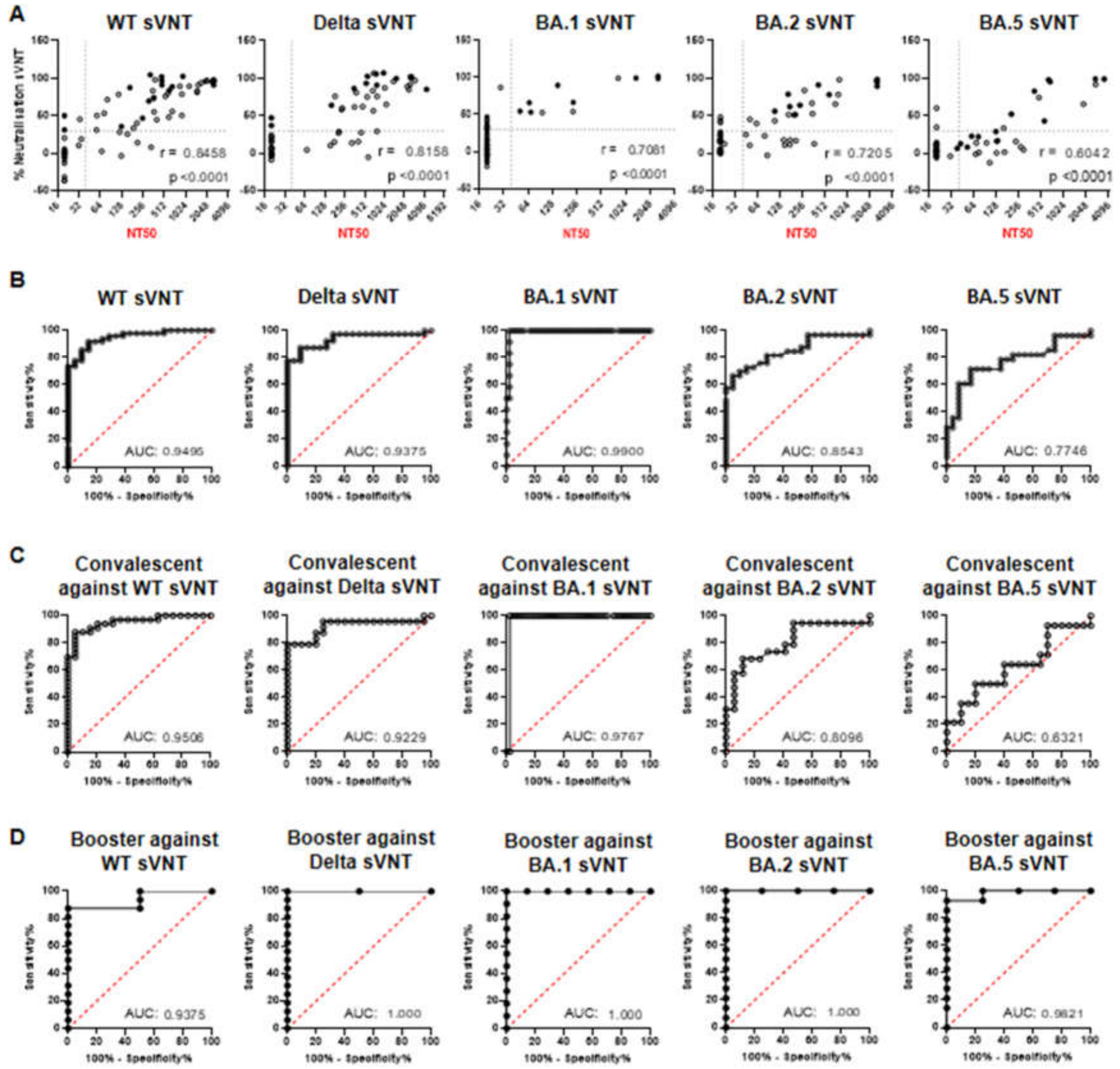

Supplement Figure S1. Correlation analysis between each VOC-sVNT and the neutralization activity (NT50) assessed by whole virus assay with the corresponding viral strain as indicated. Open circles indicate convalescent samples and closed circles indicate booster samples. The Spearman correlation coefficient (r) and p-value are indicated for each VOC-sVNT (A). ROC curve analysis of convalescent and booster samples (B) as well as convalescent (C) and booster samples (D) separately are indicated. The area under the curve (AUC) is indicated for each VOC-sVNT (B-D).

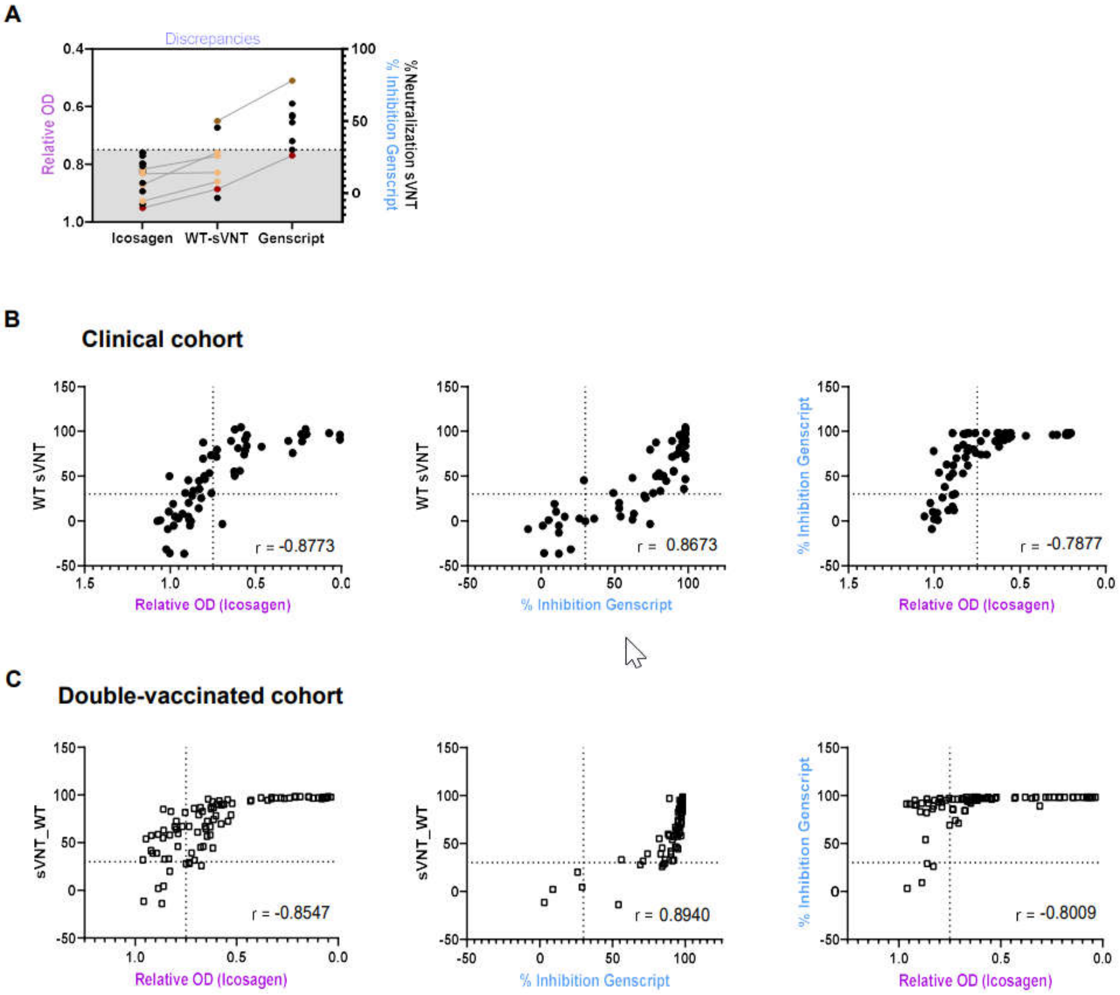

Supplement Figure S2. Comparison of clinical samples discordant in WT-sVNT, Icosagen and Genscript tests compared to WT VNT. Icosagen results are reported as Relative OD values on the left axis. WT-sVNT and Genscript percentage of neutralization and inhibition, respectively, are shown on the right axis. Black dots correspond to samples discordant in 1 out of 3 tests. Dark brown dots correspond to sera discordant in WT-sVNT and Genscript. Light brown dots correspond to samples discordant in WT-sVNT and Icosagen. Red dots correspond to sera discordant in all three tests. Grey area indicates low-no neutralization detected samples below the cutoffs for all surrogate tests (A). Correlation analysis between neutralization activity assessed by WT-sVNT, Genscript and Icosagen. Analysis of clinical samples (B) and samples of double-vaccinated individuals (C). The Spearman correlation coefficient is indicated (B-C).

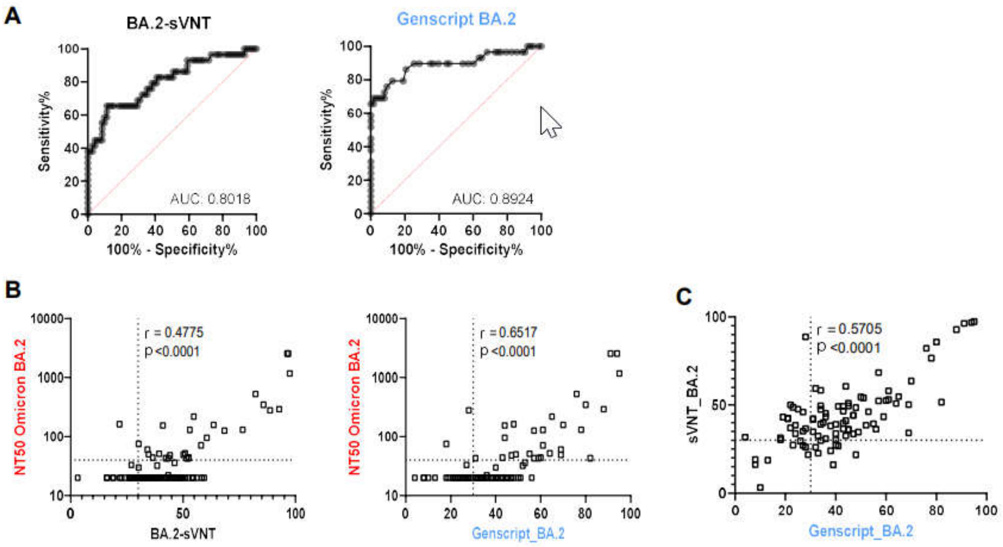

Supplement Figure S3. Performance of BA.2-sVNT in samples from double vaccinated individuals (vaccinated cohort) in comparison to BA.2 whole virus neutralization test and Genscript BA.2. Area under the curve (AUC) calculated for BA.2-sVNT and Genscript-BA.2 tested with double-vaccinated samples (A). Correlation analysis of BA.2-sVNT and Genscript-BA.2 with the neutralization activity of vaccinated samples assessed by VNT with BA.2 (B) or correlation between BA.2-sVNT and Genscript-BA.2 (C). The Spearman correlation coefficient (r) and p-value are indicated (B and C). P-values < 0.05 were considered significant. *: p<0.05; **: p<0.01; ***: p<0.001.

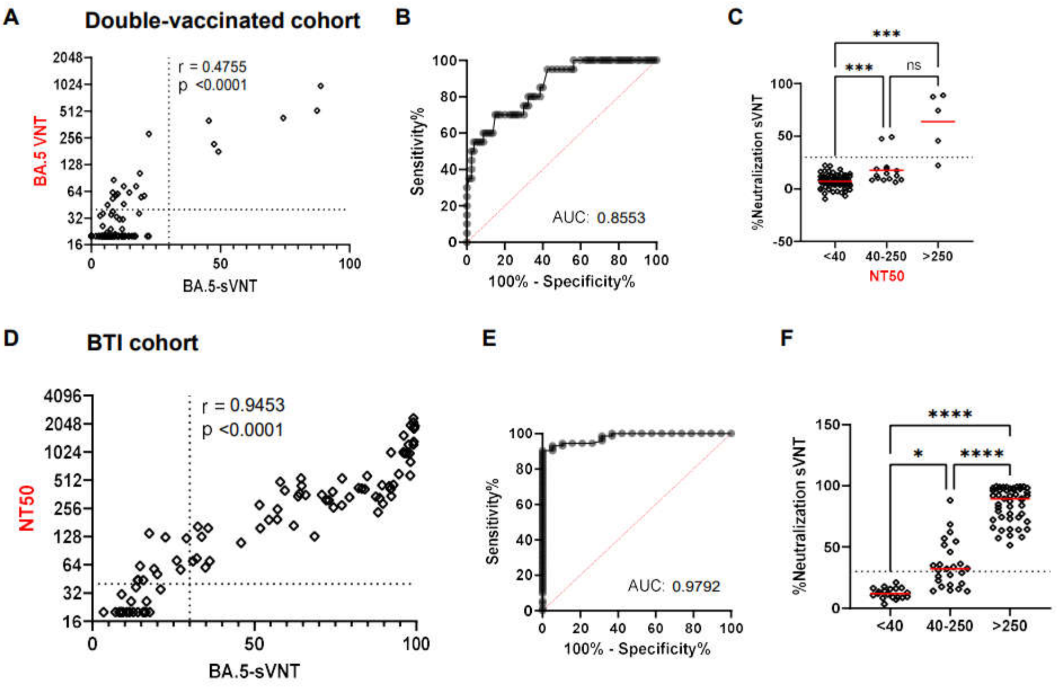

Supplement Figure S4. Performance of BA.5-sVNT in double-vaccinated samples (vaccinated cohort, A-D) and BTI samples (E-G) in comparison to BA.5 whole virus neutralization test. Correlation analysis between BA.5-sVNT and VNT using samples from the vaccinated cohort (A). ROC curve analysis of BA.5-sVNT and AUC evaluation (B). Stratification of BA.5-sVNT results in regards to NT50 titers grouped into NT50neg (samples with NT50 below 40), NT50intermed (samples with NT50 between 40 and 250) and NT50high (samples with NT50 above 250) (C). Correlation analysis between BA.5-sVNT and VNT using samples from the BTI cohort (D). ROC curve analysis of BA.5-sVNT with AUC evaluation (E). Stratification of BA.5-sVNT results in regards to NT50 titers grouped into NT50neg (samples with NT50 below 40), NT50intermed (samples with NT50 between 40 and 250) and NT50high (samples with NT50 above 25O (F).The Spearman correlation coefficient (r) and p-value are indicated (B and E). P-values were determined using Kruskal-Wallis test (C and F). P-values < 0.05 were considered significant. *: p<0.05; **: p<0.01; ***: p<0.001.
